# Understanding the Full Spectrum of Organ Injury Following Intrapartum Asphyxia

**DOI:** 10.3389/fped.2017.00016

**Published:** 2017-02-17

**Authors:** Domenic A. LaRosa, Stacey J. Ellery, David W. Walker, Hayley Dickinson

**Affiliations:** ^1^Ritchie Centre, Department of Obstetrics and Gynaecology, Hudson Institute of Medical Research, Monash University, Melbourne, VIC, Australia; ^2^Department of Pediatrics, The Alpert Medical School of Brown University, Women & Infants Hospital of Rhode Island, Providence, RI, USA

**Keywords:** birth asphyxia, cerebral palsy, HIE, creatine, spiny mouse, AKI, muscle

## Abstract

Birth asphyxia is a significant global health problem, responsible for ~1.2 million neonatal deaths each year worldwide. Those who survive often suffer from a range of health issues including brain damage—manifesting as cerebral palsy (CP)—respiratory insufficiency, cardiovascular collapse, and renal dysfunction, to name a few. Although the majority of research is directed toward reducing the brain injury that results from intrapartum birth asphyxia, the multi-organ injury observed in surviving neonates is of equal importance. Despite the advent of hypothermia therapy for the treatment of hypoxic–ischemic encephalopathy (HIE), treatment options following asphyxia at birth remain limited, particularly in low-resource settings where the incidence of birth asphyxia is highest. Furthermore, although cooling of the neonate results in improved neurological outcomes for a small proportion of treated infants, it does not provide any benefit to the other organ systems affected by asphyxia at birth. The aim of this review is to summarize the current knowledge of the multi-organ effects of intrapartum asphyxia, with particular reference to the findings from our laboratory using the precocial spiny mouse to model birth asphyxia. Furthermore, we reviewed the current treatments available for neonates who have undergone intrapartum asphyxia, and highlight the emergence of maternal dietary creatine supplementation as a preventative therapy, which has been shown to provide multi-organ protection from birth asphyxia-induced injury in our preclinical studies. This cheap and effective nutritional supplement may be the key to reducing birth asphyxia-induced death and disability, particularly in low-resource settings where current treatments are unavailable.

## Introduction

Each year approximately four million neonates become seriously deprived of oxygen (O_2_) during birth (1). There are numerous obstetric complications that can lead to an asphyxic birth. These can be loosely defined as peripartum in nature, such as placental abruption, vasa praevia (a condition where fetal blood vessels cross the external orifice of the uterus and often rupture), or a hypoxic–ischemic event at birth (2, 3). This latter category includes uterine rupture, shoulder dystocia, cord prolapse, maternal cardiopulmonary arrest, and a difficult or prolonged delivery (4). This transient but potentially catastrophic deprivation of oxygen in the intrapartum period is thought to be directly responsible for 691,000 deaths and 1.02 million stillbirths each year, making it the fifth most common cause of childhood deaths under 5 years (5–7). For those infants that do survive, the multi-organ damage that can ensue means the risk of developing severe life-long morbidities is high. Intrapartum asphyxia results in a burden of 42 million disability years (DALYs). To put this figure in context, this is twice the DALYs imposed by diabetes (8). Although it is a global issue, recent evaluations of the incidence of intrapartum asphyxia in high income countries, where adequate obstetric care is available during the peripartum period, have incidences ranging from 4.3 to 8.5% of term live births (9). This is in stark contrast to an incidence of around 23% in developing countries where women’s health care remains under-resourced and many women delivery at home, without professional assistance (10).

The unpredictable nature of the many obstetric complications that contribute to intrapartum asphyxia, in addition to the multi-organ damage associated with intrapartum oxygen deprivation (11), presents a unique set of challenges to clinical and research professionals in their endeavor to implement effective treatments for intrapartum asphyxia. Despite the burden of intrapartum-related neonatal deaths and morbidities, research investment into intrapartum asphyxia and associated morbidities remains low, potentially due to it being a condition that is most prevalent in low resource settings. When reviewing the research investment into neonatal deaths it is clear that this is an area hampered by the “10/90 gap,” meaning only 10% of research expenditure is directed toward 90% of the world’s global burden of disease (12). This should be of concern to the medical community, as the Millennium Development Goal to reduce the under-five mortality by two-thirds by 2015 was not achieved (7), and neonatal death is an increasing contributor to this category of childhood survival (13).

One cannot assume there will be a dramatic shift in research investment strategies. Hence, there is a clear need to consolidate our knowledge of the overall pathophysiology of intrapartum asphyxia; evaluate our current approaches to studying intrapartum asphyxia; and develop targeted treatments, keeping in mind the limits on application of highly sophisticated medical interventions in low resource settings. In the following review, we discuss the effects of intrapartum asphyxia on the cerebral, cardiovascular, cardiorespiratory, and musculoskeletal systems, including current treatment/management strategies and their limitations. We then provide an update on the preclinical models available for research into intrapartum hypoxia and assess treatments considered in the pipeline for clinical translation.

## The Multi-Organ Effects of Intrapartum Asphyxia

Characterization of brain injury following intrapartum asphyxia has been the focus of many basic science and clinical investigations. However, in a clinical retrospective study, Hankins et al. (14) observed that for 70% of cases, low oxygen was not the direct cause of hypoxic–ischemic encephalopathy (HIE), but rather HIE was a condition that developed secondary to renal, hepatic, and cardiac dysfunction following birth asphyxia (14). While the brain is the obvious target of interventions to allay progressive damage arising from hypoxia, oxidative stress, and inflammation at birth, the need to protect other vital organs such as the heart, kidneys, lungs, and diaphragm has received much less attention. This is made pertinent by the fact that hypothermia has not been shown to offer protection to the many other organ systems affected by birth asphyxia (15, 16), and also from the plausible possibility that HIE arises *secondarily* to poor cardiorespiratory function in the minutes and hours after an asphyxic birth. These types of studies highlight the true nature of the global oxygen deprivation associated with birth asphyxia and the need to investigate all consequential injury.

### Brain

The main clinical outcome observed in severely asphyxiated neonates is brain damage known as HIE. This varies in severity and can be categorized into one of three stages using the Sarnat method, which uses observations of the infant’s neuromuscular control, autonomic function, level of consciousness, the presence of seizures, and electroencephalographic recordings (15, 17). For infants surviving birth asphyxia and HIE in the neonatal period, the incidence of long-term poor neurological outcomes, including mental and physical disability, seizures, and cerebral palsy (CP) is high (9).

**Figure 1:**
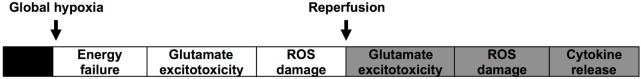
**Illustrates the commonly accepted hypothesis for the two waves of energy failure and injury in the brain after asphyxia at birth**.

At a cellular level, there are two phases or waves of brain injury following intrapartum asphyxia, as outlined in Figure 1 (18–23). The initial phase occurs during and immediately after the insult and is associated with global hypoxia. Delivery and resuscitation of the neonate then leads to apparent stabilization during the first few hours of life. However, there is a second wave of injury, which occurs from around 6 h after birth, and is associated with post-hypoxic or post-ischemic hyperemia. This induces biochemical cascades such as synaptic excitotoxicity, oxidative stress, inflammation, and cytotoxicity (18–20, 22).

Currently, the only effective treatment to reduce the adverse neurological outcomes following birth asphyxia is selective head or whole-body cooling initiated within 6 h of delivery (15). This is aimed at reducing or preventing the second wave of injury to the brain and is thought to do so by reducing metabolic rate and ameliorating the oxidative stress that ensues (15). However, the need to stabilize the infant during the first few hours of life often delays the initiation of hypothermia, which significantly reduces the effectiveness of this treatment strategy. Even without these necessary clinical delays, therapeutic hypothermia is not a wholly effective treatment. It has been reported that as many as 10 infants need to be treated to prevent one death or major disability (15, 24). Hypothermia has also been reported to have unwanted side effects such as sclerema neonatorum, hypovolemia, glucose instability, pulmonary hypertension, and multisystem organ damage if applied to unaffected neonates (16, 24, 25). Another major limitation of hypothermia as a treatment is that it is only properly deployable in tertiary health centers (8, 15, 26). However, simpler variants such as cold “hot” water bottles have been trialed in a resource-poor hospital in Uganda and shown to be a feasible approach (27). Efficacy of this treatment, however, is yet to be reported.

### Cardiac Structure and Function

It is well accepted that the physiological response to hypoxia involves the redistribution of cardiac output to maintain perfusion of vital organs, including the brain, adrenal glands, and the heart (28). However, despite the fact that blood supply to the heart is prioritized during a hypoxic event, both preclinical and clinical studies have reported deficits in cardiac function after birth asphyxia (11, 29, 30). With regard to the heart, newborns who survive intrapartum asphyxia fall into two broad categories: those who are severely ill, require urgent treatment and frequently die of heart failure during the immediate neonatal period; and those who do not have heart related complications and do not require cardiac support (14, 31). One study reported that of 46 cases of severe intrapartum asphyxia that resulted in the neonate developing HIE, 78% exhibited cardiac injury, diagnosed by elevated circulating cardiac enzymes and/or the requirement of volume support for longer than 2 h after birth (14, 15, 17). Various other clinical pathologies have also been reported, including enlargement of the heart (18–23, 32), electrocardiogram abnormalities, signs of myocardial ischemia (18–20, 22, 29, 33), arrhythmias, dysfunction of the atrioventricular valves, and sustained sinus bradycardia, as well as decreased ventricular contractility (11, 15, 29, 30). Hypotension is also a common problem encountered by neonatologists (15, 30, 34–36), often requiring treatment by volume expansion or inotropic drugs (15, 24, 25).

Despite the observation of these functional issues, the immediate and long-term structural consequences of a hypoxic birth for the heart are not well understood, with only minimal histological data reported so far. One study reported abnormalities including evidence of necrosis and phagocytosis, as well as the presence of contraction band necrosis [aggregated clumps of disorganized actin–myosin filaments (16, 24, 25, 37)] in the hearts of asphyxiated near-term lambs (8, 15, 26, 35). Human studies on non-surviving asphyxiated newborns have also reported the appearance of cardiac abnormalities (27, 32, 38), including right ventricular dilation, ventricular hypertrophy, persistent atrial shunting, and papillary muscle necrosis (39, 40). Understanding how to provide this support is hampered by a distinct lack of experimental data on the structural and functional effects of intrapartum asphyxia on the heart. Although there have been numerous studies documenting the long-term neurological outcomes for children who suffered asphyxia of varying severity during birth (11, 29, 30, 41–44), information regarding the cardiac outcomes for children with HIE and CP are not often reported. Furthermore, there appear to be no studies investigating the cardiovascular outcomes for those who suffered a mild to moderate hypoxic event at birth but who have no or little neurological disability. Consequently, there is a clear need for preclinical work using appropriate animal models that will enable these important issues to be investigated.

### Renal Structure and Function

Acute kidney injury (AKI) is a syndrome characterized by a short period of loss of the kidney’s excretory function, leading to a reduced capacity to filter blood, maintain blood volume, electrolyte levels, and acid–base homeostasis (45). AKI is usually caused by factors external to the kidney itself. In the neonate, these include premature birth, sepsis, congenital heart malfunction, and intrapartum birth asphyxia (46, 47).

Acute kidney injury has long been recognized as an almost inevitable consequence of intrapartum asphyxia, due to the shunting of blood away from peripheral organs to maintain cerebral, cardiac, and adrenal perfusion during the episode of hypoxia, thereby reducing oxygen supply to the kidney (48, 49). The renal parenchymal cells also have a limited capacity for anaerobic respiration and a high susceptibility to reperfusion injury (50). It is estimated that 50–72% of asphyxiated neonates with a 5 min Apgar score ≤6 will show signs of renal compromise (51), and studies have shown that AKI following birth asphyxia positively correlates with the risk of morbidity and mortality in asphyxiated newborns (11).

There are currently no FDA approved therapies that specifically target kidney damage after intrapartum asphyxia (52). Four, low sample number, randomized control trials have investigated the capacity for a single dose of theophylline, an adenosine receptor antagonist, in the first hour after asphyxial birth to prevent the progression of AKI (53–57). These trials were proceeded by animal studies that identified the increase in adenosine as a result of hypoxemia and renal vasoconstriction as a key contributor to the pathogenesis of AKI (58, 59). The promising results from the initial trials have led to the Kidney Disease Improving Global Outcomes (60) guidelines to suggest a single dose of theophylline to asphyxiated newborns (60); however, it is emphasized that theophylline has a narrow therapeutic window and must be well monitored as it can cause side effects, including tachycardia, hyperglycemia, vomiting, and seizures (61, 62). Experts in the field often agree that adequately powered, multicentre trials, that include assessment of long-term outcomes and theophylline as an adjunct to hypothermia are still required before the widespread adoption of this therapy (52, 57). Assessment of clinical data of babies who received hypothermia treatment alone following intrapartum asphyxia has concluded that the therapy does not reduce the rate or severity of AKI in these patients (63).

For those newborns who are accurately diagnosed with AKI, a range of management strategies can be put in place to ensure that the poorly functioning kidney does not exacerbate injury in other organs, particularly the brain (3, 47). Post-asphyxia infants presenting with oliguric AKI are at significant risk of fluid retention and hyponatremia due to decreased sodium reabsorption in the proximal tubules (64, 65). With this is mind, there is clinical recommendation to limit fluid intake in newborns with AKI; however, this is controversial as there is a fine balance between maintaining sodium retention and causing dehydration or malnutrition in these infants (3). Hyperkalemia is also of concern in newborns with AKI due to a poor capacity to produce urine and clear excess potassium from the blood. The withholding of fluids containing potassium and electrocardiographic monitoring is recommended for these babies. Imbalances in calcium and magnesium are also common complications of AKI and need to be monitored in the neonatal period (65). While these clinical guidelines can improve patient outcomes, treatment strategies focused on minimizing and repairing kidney injury are still required to combat AKI and ensure the long-term renal health of these newborns.

Furthermore, an episode of AKI in the neonatal period has also been linked to an increased risk of developing chronic kidney disease (CKD) later in life (66, 67). A recent prospective study by Mammen et al. (68) of 126 patients diagnosed with neonatal AKI aimed to assess the incidence, and risk of developing CKD in the 1–3 years after AKI. They also aimed to determine if the stage/severity of AKI at first presentation was a determinant of the development of CKD later in life. They found that 10.3% of their follow-up patients presented with CKD in the 1–3 years after AKI, as defined by the presence of albuminuria and/or decreased GFR (≤60 ml/min/1.73 m^3^). Severity of AKI (stage 3 AKI) was a potential predictor of CKD (*P* = 0.08). It should be noted that 24% of the patient population in this study were neonates, and these cases made up the majority of the AKI stage 3 population (median age 0.3 years). In addition to those who presented with CKD, 46.8% of follow-up patients were described as being at risk of developing CKD, based on a mildly reduced GFR, presence of hypertension and/or hypofiltration (68). The clinical recommendation arising from these studies is that, regardless of the severity of AKI, neonatal and pediatric patients should be monitored regularly for signs of long-term kidney damage and dysfunction, and not just in the period immediately after the episode of AKI. As early CKD is often silent, these studies strengthen the need to develop biomarkers of CKD following AKI (69). While treatment of neonatal AKI remains hindered by poor diagnostic techniques and the lack of a firm understanding of what molecular and structural changes occurs in the neonatal kidney following an asphyxic insult, a focus on prevention of injury rather than recovery may thus be a beneficial approach to develop treatments for neonatal AKI.

### Diaphragm and the Initiation of Breathing

Term neonates take their first breaths by creating a sub-atmospheric intrathoracic pressure as high as −80 mmHg, which is essential for the clearance of lung liquid, thus facilitating lung aeration and the formation of the functional residual capacity (FRC) (70–72). However, asphyxiated neonates are often unable to produce the pressures necessary to achieve this and therefore require resuscitation and subsequent mechanical ventilation (11, 33, 71). Some work has been targeted at understanding the effect of intrapartum asphyxia, together with the effects of mechanical ventilation, on the lungs. Clinical studies have reported pathologies such as hemorrhagic pulmonary edema (73) and pulmonary hypertension (33).

However, despite the crucial role it plays in lung aeration, FRC formation and the initiation of breathing at birth, the respiratory musculature has been essentially overlooked. Some recent studies have investigated the effect of different suboptimal fetal environments on the diaphragm, concluding that intrauterine inflammation caused significant impairment of diaphragm contractility in the fetal sheep (74, 75). The same group has also reported a decrease in contractility in the fetal diaphragm after maternal steroid administration (76). As for asphyxia at birth, preterm birth usually also results in the neonate requiring ventilatory support, perhaps also a result of poor diaphragm function in this respiratory insufficiency.

The mechanical effort associated with breathing after birth is high due to the high compliance of the chest wall and the relatively low compliance of the fluid-filled lung, and it is assumed that the muscle fibers of the diaphragm must be relatively resistant to fatigue. As the fetal respiratory system prepares for the transition gas exchange, the diaphragm muscle fibers undergo important biochemical and phenotypic changes to deal with this change in workload; for many species, including humans, these significant maturational changes occur *in utero*. During this time, biochemical and histochemical changes show there is a significant fast-to-slow fiber type transition, together with substantial muscle fiber hypertrophy, which begins in the immediate neonatal period and continues postnatally (14, 31, 77, 78). This highlights the perinatal period as a time when activity plays a crucial role in the functional maturation of the diaphragm, suggesting potential vulnerability during this time should an event arise which could cause damage to the diaphragm musculature and interrupt this crucial transition. Mechanical ventilation, which is known to provoke atrophy-like changes to diaphragm muscle fibers in adults (79), is therefore a significant intervention for preterm and term neonates.

The diaphragm is highly susceptible to hypoxic injury, possibly because it has a very high-energy demand. Hypoxia has also been reported to significantly reduce the diaphragm’s resistance to fatigue (14, 80–83). The mechanisms leading to this outcome are poorly understood; however, acidosis interferes with the ability of skeletal muscle to replenish ATP stores, as well as reducing the capacity of muscle fibers to utilize ATP (84). Similarly, systemic acidosis is associated with the upregulation of protein degradation pathways and result in significant structural damage and functional deficits in many different skeletal muscles (85–87). In the adult diaphragm, hypoxia reduces the ability of fibers to produce ATP using aerobic pathways, thus increasing reliance on anaerobic pathways including glycolysis and creatine phosphate degradation, a switch that has been implicated in the appearance of muscle fatigue (88–90). Hypercapnia causes structural and functional deficits in skeletal muscle, with a study by Shiota et al. (91) reporting significant reductions in functional performance and fatigue resistance, as well as alterations in the relative proportions of the different muscle fibers present in the diaphragm, and in other skeletal muscles.

Furthermore, as mentioned previously, the immense increase in ROS and RNS (reactive oxygen and nitrogen species, respectively) that follow hypoxic events results in significant oxidative stress at a cellular level. This is particularly deleterious for the diaphragm, which generates high levels of oxidants due to the continuous contractile activity of this highly oxidative muscle (88, 89, 92). Oxidative stress has been reported to increase protein degradation in skeletal muscle (93, 94), and therefore this could have significant negative effects for the newborn diaphragm as it undergoes the essential structural and biochemical changes associated with parturition and the onset of gaseous ventilation.

## Effect of Perinatal Hypoxia on Skeletal Muscle and Its Implications for CP

The body is composed of almost 40% skeletal muscle, and this tissue is not only essential for locomotion, but also plays an important role in the metabolism of proteins and amino acids for various organ systems (93). Hypoxia in adult skeletal muscle increases production of ROS, which increases the degradation of myofibrillar structural proteins (93). Exposure to hypobaric hypoxia in adults reduces maximal force production and muscle endurance as assessed by static handgrip contractions (95). Acute hypoxia increases oxidative stress and reduces mitochondrial function in mouse skeletal muscle (96), and structurally, hypoxia causes severe muscle atrophy (97). Hypobaric hypoxia results in a significant reduction in muscle mass and muscle fiber cross-sectional area (CSA) (97).

Given the obvious susceptibility of the skeletal muscle to hypoxic damage, the possibility that deficits in skeletal muscle function could be contributing to the functional motor deficits observed in survivors of intrapartum asphyxia with CP should not be overlooked. As discussed previously, CP is the most common deleterious morbidity associated with birth asphyxia. It is a condition characterized by hypotonia in the neonate (98), and children with CP often suffer from a range of neuromuscular disabilities including deficits in motor coordination, gait abnormalities and, in severe cases, upper and lower limb spasticity (99–101), and reductions of muscle endurance and peak power of the arms and legs, even in children with mild CP (102). A small study reported significant reductions in lower limb mass in children with CP (103); however, investigations into the structural changes in these muscles are lacking.

A large body of work has been dedicated to the development of treatments for the muscular symptoms of CP; for example, the use of botulinum toxin A injection to treat limb muscle spasticity, which has been shown to improve movement for children with lower limb spasticity (104–106). While the body of opinion would be that most of the muscle disability arises secondarily from damage to central pathways governing motor activity, the direct effects of mild to moderate intrapartum asphyxia on the axial muscles obviously require further consideration.

## Preclinical Models of Birth Asphyxia

A major issue faced by researchers and clinicians focusing their efforts on understanding the effects of birth asphyxia is the availability of an appropriate research model. Conventional altricial laboratory rodents such as mice and rats are commonly used to study the biochemical and pathological changes within the gray and white matter regions of the brain following intrapartum asphyxia. However, use of these species poses significant limitations due to the immaturity of their offspring at the time of birth. Therefore, any findings regarding tissue damage or alterations in normal organ development following a hypoxic episode at birth must be interpreted with caution. These would not be considered as appropriate models to study damage to peripheral organs such as the kidney after intrapartum asphyxia.

Large precocial models are much more useful in this regard as their advanced stage of development at the time of birth closely mimics the human physiological response to intrapartum asphyxia [see review in Ref. (107)]. Indeed, understanding perinatal brain injury following a hypoxic–ischemic insult was highly advanced by classic studies by Meyer et al. in the non-human primate in the 1970s (108). There is a particular ethical dilemma in the use of non-human primates, and the cost of experimentation in these species means the study of all neurological disorders in non-human primates is not always appropriate; however, they continue to be used of gain insight into the neurological outcomes of intrapartum asphyxia (109). There has also been extensive work conducted investigating the effect of birth asphyxia on the brain using both sheep and pigs. In sheep, we and others have developed and characterized a clinically relevant model of HIE in the near-term lamb (110–112), and Miller and colleagues use this model to investigate potential rescue therapies using melatonin and stem cells (113–116). Another group has used the newborn piglet to investigate the neurological effects of intrapartum asphyxia (117, 118), the effects of cooling (119) and to explore potential early biomarkers of neuronal injury (120). However, these animals are expensive, difficult to house and long-term studies, looking at the life course effect of HIE require many years.

The need for an appropriate small animal model to investigate the systemic effects of an asphyxial birth and the testing of potential interventions is met to some extent by using the spiny mouse (*Acomys cahirinis*). This is a precocial rodent native to the deserts of Africa and Middle East, where after a relatively long gestation (39 days) the neonate has completed organogenesis of major organ systems (121–124), and are fully furred, mobile and have open eyes.

Unlike other rodents, the brain of the spiny mouse is relatively well myelinated at birth (125), and the brain growth spurt in this species occurs in the days just prior to birth; this is later in gestation than the guinea pig, monkey, and sheep, but earlier than the rat and rabbit, and prior to birth as in the human. In addition, this species has a hormone profile similar to the human, with the adrenal gland producing cortisol as its major circulating glucocorticoid instead of corticosterone as seen in most rodents. The fetal spiny mouse adrenal gland is also able to synthesis the steroid dehydroepiandrosterone, making the spiny mouse perhaps one of few rodents to have a fetal-placental unit where the fetal adrenal–hepatic–placental axis plays an important role in fetal brain development during gestation, as it does in higher primates, including humans (124, 126). Finally, one of the unique intrauterine developmental aspects of the spiny mouse is the completion of nephrogenesis (the formation of the filtering units in the kidney) before birth in this species (121). This makes the study of renal hemodynamics in the early neonatal period, and the study of insults on the kidney at birth in the spiny mouse particularly relevant to the human situation.

A model of near-term intrapartum asphyxia has been produced in the spiny mouse and is characterized by global acidemia, hypoxemia, and increased plasma lactate (127). In this model, it was noted that animals that did not survive the insult (~40%) exhibited abnormal gasping behavior and were unable to establish breathing. It was therefore proposed that the respiratory muscles may be involved in this failure, and the status of the diaphragm was assessed (127). This study revealed significant structural and functional deficits in the diaphragm 24 h after birth (127). This was characterized by muscle fiber atrophy, with a 20% decrease in CSA of all three major muscle fiber types and a 27% reduction in calcium (Ca^2+^)-activated force (127) as assessed in skinned single muscle fibers. There was an increase in the expression of two pro-atrophic genes—Atrogin-1 and MuRF-1. It is therefore reasonable to assume that these deficits may be contributing to respiratory failure in these animals and thus the high mortality rate observed in this model. Since those initial studies, the spiny mouse model of intrapartum asphyxia has been used to describe molecular, structural, and functional alternations in the neonatal brain and diaphragm, kidney, gonads, and skeletal musculature, and assess the therapeutic potential of the antioxidant melatonin and intracellular energy buffer creatine (127–135).

These preclinical studies have contributed significantly to our knowledge of the longer-term impact of intrapartum asphyxia. Some of the key findings to date include neurological injury being associated with mitochondrial derangement in the cortical subplate, thalamus, and piriform cortex (133) and significant behavioral deficits in tests that assess movement and motor coordination (132). Furthermore, when the same animals were placed in an open field on postnatal day 1, and their movement was recorded, asphyxia pups traveled less distance, spent more time immobile, and made significantly fewer jumps (132). These observations suggested that these animals may have significant deficits in neuro-motor activity.

These observations were further characterized with studies aimed at directly investigating the potential effects of intrapartum asphyxia on the skeletal muscle. At 24 h of age, as was observed in the diaphragm, all three muscle fiber types showed significantly reduced size. This deficit persisted until at least 1 month of age in males, along with a significant reduction in the proportion of type I fibers and a corresponding increase in type IIb/d fibers (135). Additionally, the neonatal gastrocnemius muscle was functionally assessed *ex vivo* and, akin to the diaphragm, exhibited reduced fatigue resistance in male offspring (128, 135). It is apparent that the skeletal muscle is indeed significantly affected in the immediate neonatal period following asphyxia at birth, and these deficits are persisting into at least early adulthood in our model. Therefore, it is not unreasonable to assume that the same may be the case for human infants who survive birth asphyxia.

Finally, the first rodent model of neonatal AKI secondary to intrapartum asphyxia has been developed in the spiny mouse. Neonatal AKI was confirmed by a twofold increase in mRNA expression of *Ngal*, an early marker of kidney injury, and plasma and urinary electrolyte imbalances 24 h after insult (128). Assessment of kidney structure following intrapartum asphyxia also showed significant levels of damage and immaturity, across the renal cortex, medulla, and renal papillae. At 1 month of age, male intrapartum asphyxia offspring had a permanent loss of nephrons. Compensatory hypertrophy of remaining nephrons was apparent in this cohort, but despite this, at 3 months of age (young adult), GFR in male asphyxia offspring was significantly lower than controls (136). It is of interest that cardiac structure and function in spiny mouse neonates was unaltered by intrapartum asphyxia. Clinically, while cardiovascular dysfunction is observed in some survivors of intrapartum asphyxia, the effects on the heart are heterogenous. Therefore, the absence of an effect in surviving spiny mouse pups after asphyxia is perhaps not surprising.

## Current Treatment Strategies for Intrapartum Asphyxia

When it comes to managing the risk of a hypoxic event at birth, the main issue faced by clinicians is the unpredictable nature of intrapartum asphyxia. Most often, it is not until the woman is in labor that obstetricians become fully aware that the fetus is at risk, and by this stage there is little they can do other than to deliver the baby as quickly as possible. Furthermore, apart from hypothermia, which as previously discussed has limited success, there are no other effective treatments for preventing the two waves of energy failure that ensues following an asphyxic birth.

A number of antenatally applied treatments that aim to prevent intrapartum hypoxic *brain* damage have been proposed and include the use of ascorbic acid, tetrahydrobiopterin, phenobarbital, *N*-acetylcysteine (NAC), xenon, and argon. However, these have either provided disappointing results (ascorbic acid and xenon), require specialized ventilatory equipment (xenon and argon), revealed unfavorable safety profiles (NAC), or, while showing promise (e.g., allopurinol, melatonin, and argon), currently lack full clinical evaluation, including their capacity to target hypoxic injury in organs other than the brain. Pharmacokinetically, all of these agents have a short duration of action and cannot be expected to provide a benefit lasting through birth into neonatal life unless their administration continues throughout the entire ante-, intra-, and postpartum periods.

The gold standard would be a safe treatment that could be administered maternally before birth to provide protection in the event of a hypoxic episode during labor. Such a treatment should be cheap and easily administered so that it could be made available in the areas where the incidence of birth asphyxia is highest. It must also have the capacity to minimize the initial energy failure that occurs at the time of the insult, as well as having potent antioxidant capabilities to reduce the oxidative stress that ensues in the period immediately after birth. This would reduce or prevent the second wave of energy failure and oxidative stress and prevent the multi-organ injury that results. It will also need to be benign should intrapartum hypoxia not arise.

Elsewhere, we have proposed that antenatally administered creatine can provide the fetus and neonate with an additional pool of anaerobic energy during severe hypoxia, and it is a treatment that will provide protection not only to the brain but also to the other major organs that often damaged by “oxygen starvation” at birth (137). Creatine, as phosphocreatine, provides a phosphate group for the anaerobic regeneration of ATP from ADP (138). This system is crucial in maintaining intracellular energy homeostasis, serving as both a spatial and temporal energy buffer in cells with high and fluctuating energy demands such as striated muscle and neurons of the central nervous system (139). In addition to this role, it is also known to have other important properties. The conversion of creatine to phosphocreatine utilizes a proton, reducing intracellular acidity and thus maintaining acid–base balance (140). It has also been reported to have antioxidant properties in studies investigating the effect of hypoxia on the skeletal muscle (140, 141) and brain (133). These studies hypothesized that increasing total intracellular creatine before birth (creatine + phosphocreatine) would protect the fetus from the damaging effects of birth asphyxia by providing an additional, anaerobic source of ATP when oxidative phosphorylation fails due to the hypoxic insult. The antioxidant properties of creatine could also act to reduce the oxidative stress associated with the global hypoxia–ischemia that ensues. Using our spiny mouse model of birth asphyxia, we have shown that supplementing the maternal diet with 5% creatine monohydrate for a period of 19 days before delivery, caused significant creatine loading in the fetal brain, heart, kidney, liver, and diaphragm muscle (127, 131). These studies also reported a significant reduction in neonatal mortality rate after birth asphyxia (127, 131) and was protective for the brain (133), diaphragm (127), kidney (128, 136), and axial skeletal muscles (134, 135).

Despite these interesting and promising results, to date there have been no clinical studies investigating the potential for creatine to prevent birth asphyxia-induced injury. However, this was the subject of a recent review in which the limited amount of data available was highlighted (142). A full Cochrane has also been published (142); however, due to the lack of data for human pregnancy, this brought to light the need for clinical translation of this potentially life-saving treatment. If antenatally administered creatine proves to be effective and safe, it would be a cheap and readily available prophylactic treatment that could be recommended to all pregnant woman as a safeguard in the event of intrapartum hypoxia and neonatal asphyxia, thus reducing the high mortality, morbidity, and financial burden associated with this common and devastating event, in much the same way that folate supplementation has prevented neural tube defects. In the 1960s, Smithells and Hibbard discovered that women who gave birth to children with serious birth defects, notably neural tube defects, had evidence of impaired folate status. Some 30 years later, randomized controlled trials showed that they were right, and folate supplementation was effective at protecting against neural tube defects (143). In addition to our extensive evidence from animal studies that support a benefit for the fetus of increasing tissue creatine content above normal levels, we have recently shown that women who deliver small babies have lower urine creatine concentrations (144), suggesting that inadequate creatine availability may impact fetal growth. We are currently testing this hypothesis in a large prospective cohort of low risk pregnant women.

## Summary

Reducing the under-five mortality rate by two-thirds by 2015 was a Millennium Development Goal that unfortunately was not achieved (7). While advancements in obstetric monitoring and therapies such as hypothermia have helped reduce the rate of intrapartum-related brain damage and death significantly in the last decade, more needs to be done to address problems associated with the more extensive (multi-organ) compromises that may affect these infants. This has significant economical and emotional burdens that have not been adequately addressed and is of particular relevance for developing countries where medical resources may be limited. Severe hypoxia at birth is essentially a cardiorespiratory problem, and while brain damage has received the most attention, the global nature of the hypoxic–ischemic insult and myriad of biochemical disruptions that follow cause significant injury to many organ systems, as outlined in this review. Year 2016 marks the beginning of new global Sustainable Development Goals, with the aim to have the “Under-5” mortality rate below 25 per 1,000 live births by 2030 (145). As we embark on this new period it is important that we engage in preclinical and clinical studies designed to directly address the multi-faceted outcomes of intrapartum asphyxia. Careful consideration to appropriate animal models is required to ensure that the limited resources invested in this research provide the maximum, and most relevant, outcomes that further our understanding of injury to all the major organ systems in the neonates. The development of adjuncts to hypothermia and new therapies should be carefully considered for their applicability in the developing world if we are to truly combat the global burden of intrapartum asphyxia. Furthermore, experimentally, we are duty-bound to provide evidence for the effectiveness of new therapies beyond the neonatal period, i.e., into infancy, childhood, and the adult stages of life.

## Author Contributions

All authors contributed to writing and drafting this review.

## Conflict of Interest Statement

The authors declare that the research was conducted in the absence of any commercial or financial relationships that could be construed as a potential conflict of interest.
